# Evaluation of the Management of Patients with Detectable Viral Load after the Implementation of Routine Viral Load Monitoring in an Urban HIV Clinic in Uganda

**DOI:** 10.1155/2019/9271450

**Published:** 2019-12-15

**Authors:** Nsumba Steven Mark, Musomba Rachel, Arvind Kaimal, Mubiru Frank, Tibakabikoba Harriet, Lwanga Isaac, Mohammed Lamorde, Castelnuovo Barbara

**Affiliations:** Infectious Diseases Institute, Makerere University College of Health Sciences, Kampala, Uganda

## Abstract

**Objective:**

To describe the clinical decisions taken for patients failing on treatment and possible implementation leakages within the monitoring cascade at a large urban HIV Centre in Kampala, Uganda.

**Methods:**

As per internal clinic guidelines, VL results >1,000 copies/ml are flagged by a quality assurance officer and sent to the requesting clinician. The clinician fills a “decision form” choosing: (1) refer for adherence counselling, (2) repeat VL after 3 months, and (3) switch to second line. We performed data extraction on a random sample of 100 patients with VL test >1,000 copies/ml between January and August 2015. For each patient, we described the action taken by the clinicians.

**Results:**

Of 6,438 patients with VL performed, 1,021 (16%) had >1,000 copies/ml. Of the 100 (10.1%) clinical files sampled, 61% were female, median age was 39 years (IQR: 32–47), 81% were on 1^st^-line ART, 19% on 2^nd^-line, median CD4 count was 249 cells/*µ*L (IQR: 145–390), median log_10_ VL 4.42 (IQR: 3.98–4.92). Doctors' decisions were; refer for adherence counseling 49%, repeat VL for 25%, and switch to second line for 24% patients. Forty-one percent were not managed according to the guidelines. Of these, 29 (70.7%) were still active in care, 7 were tracked [5 (12.2%) lost to program, 2 (4.9%) dead] and 5 patients were not tracked.

**Conclusion:**

Despite the implementation of internal systems to manage patients failing ART, we found substantial leakages in the monitoring “cascade”. Additional measures and stronger clinical supervision are needed to make every test count, and to ensure appropriate management of patients failing on ART.

## 1. Introduction

Remarkable progress has been made in the scale-up of antiretroviral therapy (ART) in low and middle-income countries (LMICs). Currently, approximately 21.7 million people are receiving ART globally, including 15 million people in sub Saharan Africa [1, 2].

In order for HIV treatment to be effective in restoring immune-functionality, ART should control viral replication and patients should achieve viral suppression. Therefore, periodic viral load (VL) testing is considered the gold standard approach for ART monitoring in HIV positive patients and in countries where VL testing is available. The most common cause for not achieving viral suppression is poor adherence, however, suboptimal drug concentrations due to malabsorption or drug–drug interaction, and transmitted drug resistance can ultimately contribute to viral replication [3].

In the 2013 consolidated ART guidelines [4], WHO recommends monitoring ART efficacy using viral load (VL) testing performed at six months following initiation, and annually afterwards. The WHO cut off for viral failure is 1,000 copies due to limited transmission [5] and slow disease progression [6] when VL is maintained under this cut-off. For patients with VL >1,000 copies/ml, intensified adherence counselling (IAC) is recommended. Here patients receive monthly adherence counselling sessions for three months followed by a confirmatory viral load testing at 3–6 months after completion of IAC cycles.

Delayed switching to second line treatment in patients with treatment failure increases risk of morbidity and mortality [7, 8]. Additionally, achieving and maintaining viral suppression is key to substantially decrease HIV transmission [9]. However, a recent survey by WHO in LMICs found that only 20% of patients on ART receive VL testing [4]. In addition, early reports on the use of VL monitoring highlight that a substantial proportion of patients on first-line ART with confirmed virological failure are not being appropriately switched to second-line ART [1, 10].

We investigated clinical decision-making and subsequent management of a sample of patients with VL >1,000 copies/ml after the implementation of routine VL monitoring at a large urban HIV Centre in Kampala, Uganda.

## 2. Methods

### 2.1. Settings

The Infectious Diseases Institute (IDI), Makerere University is a center of excellence [11] for HIV care and management located within Mulago Hospital in Kampala Uganda. The Prevention, Care and Treatment (PCT) Programme at IDI provides high quality care and treatment to an average of 8,000 active HIV/AIDS patients. Special clinics for different HIV infected populations, such as sero-discordant couples, adolescents, older adults, pregnant mothers, MARPs and those with noncommunicable diseases are all in place. IDI plays a role as a referral Centre for more complicated cases within the national referral system. Since 2004, IDI has provided ART free of charge to patients in line with WHO and Uganda Ministry of Health ART guidelines [4]. Up to December 2014, VL testing was performed “only for patients with suspected treatment failure based on clinical and immunological criteria. Subsequently, centralized VL testing commenced at the Central Public Health Laboratory (CPHL) and routine VL testing was adopted into the National ART program [4].

**Figure 1:**
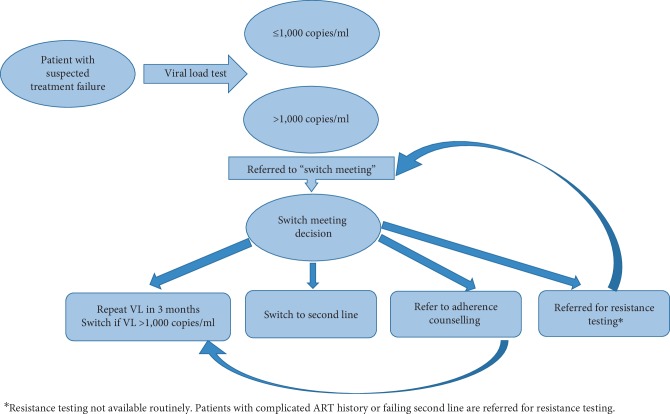
The figure demonstrates all the steps the patients with detectable viral load underwent, Via switch-meeting-including ref-feral for adherence counselling, repeat VL after 3 months of IAC, switching to 2nd line treatment and referral for resistance profile.

In prevailing clinic guidelines in 2015, VL results >1,000 copies/ml were flagged by a quality assurance officer and managed through a multidisciplinary (clinicians, nurses, counselors and pharmacists) weekly “switch-meeting”, previously described [12]. At these meetings, a decision was taken on the future management of the patients including (1) adherence counseling and repeating VL, (2) immediate switch to second line ART, (3) resistance profile test, and (4) further investigation or a combination of any of the above. The decisions were usually based on psycho-social aspects, adherence history, history of treatment interruption, history of drug–drug interaction and concomitant opportunistic infections. Resistance testing was usually reserved for patients with no obvious explanation for treatment failure, counselling was offered to patients with short periods of suboptimal adherence while an immediate repetition of VL may have been triggered by a concomitant opportunistic infection and the need of fast switch to a functioning ART regimen. Details of the “viral failure” pathway are shown in Figure 1.

### 2.2. Study Design and Analysis

This was a retrospective study of 100 randomly sampled HIV infected patients on ART who had a VL results >1,000 copies/ml from January to August 2015 at IDI.

Data from patient's clinical charts and the clinic electronic database [13] were validated and extracted, and it included: demographic data, VL and CD4 counts, WHO staging, ART regimens and duration, actions taken and switch dates.

### 2.3. Statistical Data Analysis

Actions taken by the clinicians for each patient were summarized.

Statistical analysis was performed using Stata version 12.2 (Stata Corporation, College Station, Texas, USA).

### 2.4. Ethical Statement

The study and protocol for retrospective use of routinely collected data at IDI was reviewed and approved by the ethics and protocol review committees of Makerere University, Faculty of Medicine Research and Ethics Committee (approval number: REC REF No. 2009-120) and the Uganda National Council for Science and Technology (approval number: HS 683). Patient's information was analyzed after anonymization by removal of unique personal identifiers and as such, they did not provide written or verbal consent as per the protocol procedures.

## 3. Results

**Figure 2:**
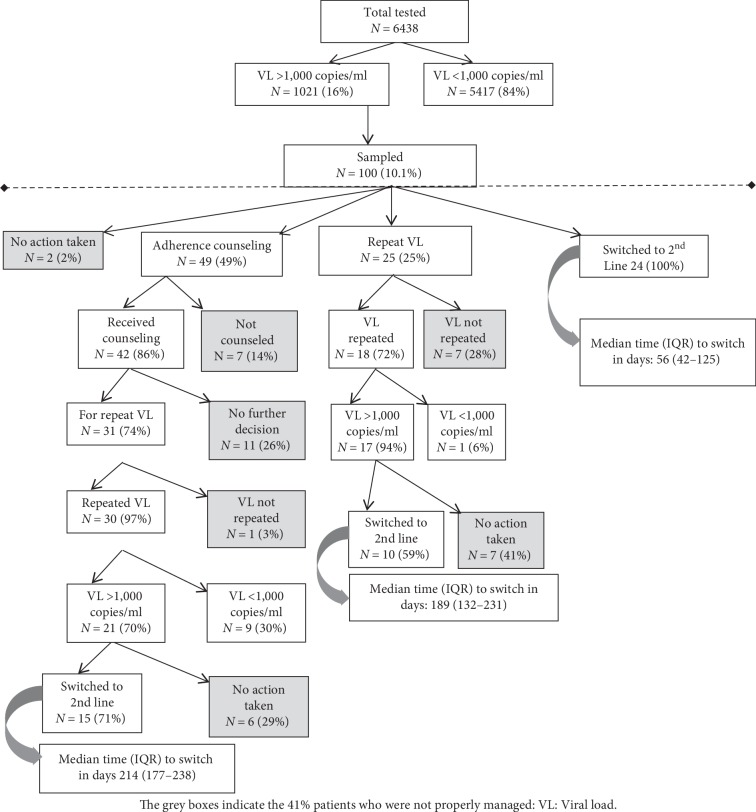
Demonstration of the number of patients who had detectable viral loads and the actions taken for the 100 patients following the IDI treatment failure pathway algorithm including the proportions of patients who were referred for adherence counselling, VL retesting, 2nd line switches and those who had no action taken.

Between January and August 2015, 6,438 patients had their VL done, 1,021 (16%) had detectable viral loads (VL) (>1,000 copies/ml). Of the sampled 100 (10.1%) patients, majority (61%) were female, the median age was 39 years (IQR: 32–47), the median CD4 count was 249 cells/*µ*L (IQR: 145–390), the median log_10_ VL 4.42 (IQR: 3.98–4.92) and a majority (81%) were on 1^st^-line ART.  Figure 2 summarizes the actions taken for the 100 patients following the IDI treatment failure path-way algorithm. Of the 100 sampled patients discussed in the switch meeting, 24 patients were directly switched to second line ART after a median time of 56 days (IQR 42–125) from the time of the detectable viral load, 25 were recommended for a repeat viral load test after 3 months, and 49 patients were referred for at least one intensive adherence counseling session before viral load retesting, while for 2 no action was taken.

Of the 25 patients who were recommended for a repeat viral load, 18 (72%) had the test repeated, of which 17 (94%) had a VL >1,000 copies/ml and of these 10 (59%) were switched to 2^nd^ line ART after a median time of 189 days (IQR: 132–231).

Forty-two (86%) of the 49 patients who were referred for adherence counseling, were counseled and after 3 months, 31 of them (74%) were recommended for a repeat VL; 30 (97%) had their VL retested among which 21 (70%) had VL >1,000 copies/ml; 15 (71%) were also switched to 2^nd^ line after a median time of 214 days (IQR 177–238).

As shown in the greyed boxes in Figure 2, a total of 41% of the patients were not managed according to the clinic guidelines, and of these, 29 (70.7%) patients were still active in care. Following tracking of seven of these patients, 5/41 (12.2%) were found to be lost to program, 2 (4.9%) dead; 5 patients were not tracked.

## 4. Discussion

In this study we evaluated the management of patients with suspected treatment failure as indicated by their detectable viral loads in our program. In 59% of the cases, patient management adhered to the prevailing clinic guidelines despite introduction of a quality assurance officer to track and coordinate detectable VL results. However, prior to the implementation of routine VL monitoring, we conducted a similar analysis on patients who receive clinician driven VL testing due to a suspicion of treatment failure and we found high (96%) adherence to the guidelines [12].

Our study likely indicates that introduction of routine VL testing increased volumes of patients with detectable VL to manage which placed a strain on the clinic system for managing patients with treatment failure. Notably, we observed considerable delays in switching patients to second line, but also for patients that were correctly managed; this was mainly due to delays in discussing the cases at the weekly switch meeting, due to the sudden increase in numbers of patients detected, with viral failure.

Our study contributes to the literature by delineating the exact steps within the cascade where nonadherence to guidelines occurs and that can be used to develop interventions. Although no formal assessment was conducted to ascertain the relative contribution on possible factors responsible for the leakages in our monitoring cascade, we believe that introduction of a quality control system to ensure timeliness in implementing patient management decisions along the cascade would be beneficial.

## 5. Conclusion

Substantial leakages and delays in the cascade for managing patients with detectable VL still exist. In order to make every patients' VL test count, additional measures including substantial investment in strengthening internal health quality control systems and structures as well as capacity building among medical staff to strengthen clinical care decisions and closer monitoring of patients for timely decision making along the care cascade should be implemented.

## Data Availability

We have no data provided as part of the supplementary information to this manuscript.

## References

[1] 
Haas A. D., Keiser O., Balestre E. (2015). Monitoring and switching of first-line antiretroviral therapy in adult treatment cohorts in sub-Saharan Africa: collaborative analysis. *The Lancet HIV*.

[2] WHO (2015). *Antiretroviral Therapy (ART) Coverage Among All Age Groups [Global Health Observatory (GHO) Data]*.

[3] Nachega B. J., Marconi  C. V., van Zyl  U. G. (2011). HIV treatment adherence, drug resistance, virologic failure: evolving concepts. *Infectious Disorders-Drug Targets (Formerly Current Drug Targets-Infectious Disorders)*.

[4] WHO (2016). *Consolidated Guidelines on the Use of Antiretroviral Drugs for Treating and Preventing HIV Infection: Recommendations for a Public Health Approach*.

[5] Loutfy M. R., Wu W., Letchumanan M. (2013). Systematic review of HIV transmission between heterosexual serodiscordant couples where the HIV-positive partner is fully suppressed on antiretroviral therapy. *PLoS One*.

[6] Opportunistic infections project team of the collaboration of observational HIV epidemiological research in Europe , Mocroft A., Reiss P. (2010). Is it safe to discontinue primary Pneumocystis jiroveci pneumonia prophylaxis in patients with virologically suppressed HIV infection and a CD4 cell count< 200 cells/*µ*L. *Clinical Infectious Diseases*.

[7] Mgosha P. C. (2017). Barriers to Switching Patients to Second-Line Antiretroviral Treatment Among Clinicians in Tanzania.

[8] Omari H. R. (2014). Second-Line Antiretroviral Therapy in Northern Tanzania: The University of North Carolina at Chapel Hill.

[9] Lecher S. (2016). Progress with scale-up of HIV viral load monitoring—seven sub-Saharan African countries, January 2015–June 2016. *MMWR Morbidity and Mortality Weekly Report*.

[10] Jobanputra K., Parker L. A., Azih C. (2014). Impact and programmatic implications of routine viral load monitoring in Swaziland. *Journal of Acquired Immune Deficiency Syndromes*.

[11] Ronald A., Kamya M., Katabira E., Scheld W. M., Sewankambo N. (2011). The infectious diseases Institute at Makerere University, Kampala, Uganda. *Infectious Disease Clinics*.

[12] Barbara C., Mark N., Rachel M. (2015). Strengthening the viral failure pathway: clinical decision and outcomes of patients with confirmed viral failure in a large HIV care clinic in Uganda. *Journal of Acquired Immune Deficiency Syndromes*.

[13] Castelnuovo B., Kiragga A., Afayo V. (2012). Implementation of provider-based electronic medical records and improvement of the quality of data in a large HIV program in sub-Saharan Africa. *PLoS One*.

